# An observational cohort study of longitudinal impacts on frailty and well‐being of COVID‐19 lockdowns in older adults in England and Spain

**DOI:** 10.1111/hsc.13735

**Published:** 2022-01-28

**Authors:** Ian W. Garner, Sandra Varey, Esperanza Navarro‐Pardo, Calum Marr, Carol A. Holland

**Affiliations:** ^1^ Division of Health Research Lancaster University Lancaster UK; ^2^ Lancaster Centre for Ageing Research (C4AR ) Lancaster University Lancaster UK; ^3^ Department of Developmental and Educational Psychology Universitat de Valencia Valencia Spain

**Keywords:** COVID‐19, COVID‐19 risk, frailty, health behaviours, lockdown, longitudinal analysis, older people

## Abstract

To reduce the spread of COVID‐19, governments initiated lockdowns, limiting mobility and social interaction of populations. Lockdown is linked to health issues, yet the full impact on health remains unknown, particularly in more vulnerable groups. This study examined the impact on frailty and outcomes in high and low COVID‐19 risk older adults. We examined health‐related behaviours and support resources participants used during lockdown(s). Lockdown impacts in two countries were compared across four time points to examine impacts of different rules.

We recruited 70 participants (aged >70 years) in England and Spain. Participants were allocated to higher or lower COVID‐19‐risk groups based on UK NHS guidelines. They completed assessments for frailty, quality‐of‐life, loneliness, exercise frequency and social interaction, coping resources and perception of age‐friendliness of their environment. The four assessments took place over a 7‐month period.

Frailty was highest at Time 1 (most severe lockdown restrictions) and significantly higher in the Spanish group. It was lower at Time 3 (lowest restrictions), but did not continue to reduce for the English participants. Perceptions of the age friendliness of the environment matched these changes. Coping resources did not mitigate changes in frailty and outcomes over time, but more frequent physical activity predicted more reduction in frailty. Lockdown had a negative impact on frailty, increasing risk of adverse events for older people, but recovery once lockdowns are eased is evidenced. Further research is required to consider longer term impacts and methods to mitigate effects of lockdown on health.

**What is known about this topic**

- Older adults have the highest risk of suffering adverse effects from COVID‐19.
- Lower physical activity and increased loneliness and isolation increase frailty, and so lockdowns may impact on frailty. Increased frailty is expected to increase susceptibility to adverse COVID‐19 outcomes.

**What this paper adds to this topic**

- COVID‐19 research is largely cross‐sectional, whereas the current study demonstrates change over time in frailty and well‐being.
- Impact of restrictiveness of lockdown(s) on older adults’ well‐being and frailty is demonstrated.
- While use of coping resources varied with lockdown stages, only perceived age friendliness of the environment and physical activity mitigated the impact of lockdown on well‐being and frailty.

## 1 INTRODUCTION

There have been over 4 million worldwide COVID‐19‐related deaths, with the age‐specific fatality rate rising from 0.01% at age 25, to 4.6% at age 75 and 15% at age 85 years (Levin et al., 2020). Those most susceptible to severe reactions to COVID‐19 are adults aged 65+ (Williamson et al., 2020), especially those living with respiratory issues and/or morbid obesity (Onder et al., 2020). Medical conditions such as dementia, diabetes, COPD, pneumonia, depression, atrial fibrillation and hypertension are also risk factors for COVID‐19‐related hospitalisations and mortality (Atkins et al., 2020).

From March 2020, Spanish and UK governments initiated national lockdowns to reduce COVID‐19 transmissions. Exercise was allowed in the UK, but not in Spain, and the UK emphasised the risk to people aged 70+. The effects of lockdown on older adults’ health and well‐being remains unknown, and there is minimal research assessing the effects of lockdown on frailty. To examine this, different phases of lockdown (i.e. different levels of restrictions) and differences between countries present a useful opportunity to examine associations of effects with severity and phases of restrictions. The two countries chosen (England and Spain) both had high COVID‐19 infection rates at the outset of the study.

Frailty is defined as a state of increased vulnerability to negative outcomes when exposed to adversity such as illness or falls (Clegg et al., 2013). Therefore, outcomes of COVID‐19 infection are more severe for frail older adults (Hewitt et al., 2020; Ma et al., 2020; Petermann‐Rocha et al., 2020). Physical and social inactivity caused by lockdown is likely to have an immediate and lasting effect on older adults’ health, with pre‐pandemic evidence linking low exercise and social engagement to increased risk of frailty, cognitive decline, and dementia (Apóstolo et al., 2018; Gale et al., 2018; Livingston et al., 2020; Oliveira et al., 2020). Carriedo et al. (2020) showed that older adults who met the global recommendations on vigorous and moderate‐vigorous physical activity had higher resilience, positive affect and lower depressive symptoms than those who did not. However, only 32.7% of Spanish older adults continued during lockdown, while 65.7% reported doing less physical activity (Rodríguez‐Gonzalez et al., 2020). It is therefore important to understand how lockdown restrictions influenced frailty levels and vulnerability to infection over the course of the pandemic.

Research on physical health or frailty impacts of COVID‐19 is less prevalent in the literature than on mental health. After one month of lockdown, mental distress in the UK general population had risen by 7.4% (Pierce et al., 2020). In Spain, people showed high rates of depressive symptomology (18.7%), anxiety (21.6%) and post‐traumatic stress symptoms (15.8%; González‐Sanguino et al., 2020), with rates of anxiety, depression and stress increasing from the first to second month of lockdown (Planchuelo‐Gómez et al., 2020). Additionally, 24.7% of Spanish people reported moderate or severe psychological impact while 48.8% showed mental health problems (Parrado‐Gonzalez and León‐Jariego, 2020). Whilst these findings provide some understanding regarding implications of lockdown, they are based on general population samples. Specific study of older populations is needed.

Lockdown effects could be severe in older adults prefer face‐to‐face socialising as opposed to technology use, the main form of interaction in lockdown (Bell et al., 2013), suggesting that older adults have fewer avenues of communication. This may impact on well‐being through increased feelings of loneliness and social isolation (Gombault, 2013). The issue is illustrated by Fang et al. (2018) who demonstrated that using the internet for communication was associated with better life satisfaction and lower depression amongst older adults. Additionally, older adults are more likely to live alone and consequently become socially isolated during lockdown. However, some studies have found age to be related to lower psychological risk during the lockdown; García‐Fernández et al. (2020), with Rossi et al. (2021) suggesting that resilience mediated the effects of stressful COVID‐19‐related events on depressive and anxiety symptoms and perceived stress in older groups. Nevertheless, there is evidence that stress, anxiety and depression were higher in older‐old adults generally and those with chronic illness (Picaza‐Gorrochategi et al., 2020). Furthermore, Di Gessa and Price, (2021) reported that older adults who were classified as being at greater risk from COVID‐19 due to pre‐existing health conditions were more likely to have poorer health outcomes. Therefore, we will examine the impact of lockdowns on people aged over 70, purposively sampling to include people living with severe chronic illnesses.

Despite these findings, research has not considered how to mitigate adverse effects of lockdown. With repeated easing and reinstating of restrictions in response to the COVID‐19 pandemic, it is reasonable to expect that restrictions will be enforced in countries on a long‐term basis. Therefore, we will consider what factors may mitigate the effects of lockdown on older adults, with coping resources, and health behaviours such as physical and social engagement identified as candidate protective factors. The person‐fit of a person's environment is also important to consider, whereby both physical and social attributes of one's home and community may contribute to the quality of life, particularly in lockdown situations where community support for older people may increase but access to local facilities would decrease. Therefore, the role of the age friendliness of the environment from the person's point of view will also be considered (Garner & Holland, 2019).

The objectives of this study were to assess potential changes in frailty and mental well‐being in older adults across a 7‐month period through lockdown variations to determine longer‐term impacts of lockdowns and how frailty may change as the pandemic continued. We focused on a multidimensional, holistic approach to frailty and its measurement (Garner et al., 2020; Gobbens et al., 2012). We also considered if coping resources mitigate any increase in frailty or decline in well‐being associated with lockdown, and how frequency of physical activity and experienced social isolation influenced the trajectory of change in frailty, loneliness, quality of life and perceptions of environmental age‐friendliness (AFEAT).

### 1.1 Hypotheses

1. Measures of frailty, loneliness, social isolation, quality of life and perceptions of environmental age‐friendliness will vary in relation to the severity of COVID‐19 lockdown in repeated measures analyses over a 7‐month period from the first COVID‐19 lockdown.
2. These variations will be different in the two countries where there are differences in lockdown severity.
3. These variations will differ according to level of risk related to the presence of significant chronic diseases.
4. Potential modifying factors–coping resources, physical activity, and social engagement/isolation–will be different under the different lockdown severities.
5. These factors will modify adverse effects of lockdown such as frailty,^1^ loneliness, quality of life and AFEAT.

## 2 METHODS

This report has been written with reference to the STROBE checklist (Strengthening Reporting of Observational Studies in Epidemiology; von Elm et al., 2007).

### 2.1 Study design

A longitudinal cohort design was used for within participants comparisons across four time points at 6‐week intervals, starting in May 2020 and ending October 2020. Between‐participants comparisons were also possible between participants in England and Spain, and between groups of different levels of COVID‐19 risk.

### 2.2 Setting

Participants were recruited in the North West and Midlands of England and in Valencia in Spain. While lockdowns were similar, the first lockdown (Time 1) was stricter in Spain than in the UK with people not allowed out for a walk in Spain and UK residents encouraged to exercise daily. In the region in England where participants were located, the tiers system introduced meant that restrictions were increasing at Time 4.

### 2.3 Participants

Participants were required live in their own home or sheltered accommodation and be aged over 70. They needed to demonstrate Mental Capacity to consent, with all researchers trained in the use of the Mental Capacity Act (2007). Recruitment was conducted by advertising on the University ageing research centre website, local senior groups and via personal approach to people who had taken part in a previous study for people living with chronic illness, and indicated they would be willing to do so again.

Sample size was determined using sample size analysis for four repeated measures with power of 0.8 and alpha of 0.05, for a medium effect size. This gave a required sample size of 24 (GPower^®^).

Fifty participants were recruited in England (33 female, 17 male, aged 70–85 years) and 20 in Spain (14 female, 6 male, aged 72–85 years). Of the total, 24 (21 in England, 3 in Spain) were considered ‘high risk’ (i.e. highly vulnerable to severe effects of COVID‐19 and should ‘shield^2^’, 15 female, 9 male, aged 70–85 years). This was determined by asking participants if they had received a letter from their GP or by making a judgment based on their declared diagnoses, referring to UK NHS lists of high‐risk conditions (NHS, 2020). The remaining 46 participants were considered ‘low risk’ (32 female, 14 male, aged 70–85 years).

Of the initial 70 participants, 65 completed assessments at all time points. The remaining five participants could not be contacted for all assessments.

### 2.4 Variables and measures

A frailty profile consisting of physical, psychological, social, and environmental domains, derived from the Tilburg Frailty Indicator (Gobbens et al., 2012), and the Community Frailty Index (Garner et al., 2020), was used to assess frailty (range 0–1, higher scores indicating greater frailty severity). The 10‐item Age‐Friendly Environment Assessment Tool (AFEAT; Garner & Holland, 2019) was used to assess participants’ perceptions of how well suited their environment is to meet their needs; range 10–50, higher scores indicating participants perceived their environment to be more age‐friendly. Quality of Life (QoL) was assessed using the Control, Autonomy, Self‐Realisation and Pleasure 12‐item (CASP12; Wiggins et al., 2008); range 12–48, higher scores indicating better QoL. A section of the Adult Social Care Outcomes Toolkit (ASCOT) was used to assess social isolation (assessing number of close friends, family and frequency of interaction with them–higher score indicates less social isolation; Netten et al., 2012). Loneliness was assessed using the shortened UCLA Loneliness Scale (Russell et al., 1980); range 4–12, higher scores indicate lower feelings of loneliness.

Participants were asked how many days a week they engaged in 30+ minutes of exercise. Responses were coded as an ordinal variable (1 = 0–1 days, 2 = 2–3 days, 3 = 4 days, 4 = 5–7 days). Participants were asked whether any of a list of nine potential factors had helped them to cope during lockdown (family, friends, NHS, watching TV, exercise, community groups, specific organisations, shopping and humour) and were invited to add others to the list. Level of education was coded as an ordinal variable (1 = None/Primary Education, 2 = Secondary Education to 16 years of age, 3 = Secondary Education to 18 years of age, 4 = Higher Professional or University Education). The current occupation was used as a proxy measure of socioeconomic status. If participants were retired, they were asked to state their profession/main job during their working career. Occupations were coded 1–8 according to the National Statistics Socio‐economic classification system (The National Statistics Socio‐Economic Classification, 2005). Participants were assessed in their native language by native speakers of that language, using translations of the largely English scales into Spanish for the Spanish group.

The assessments combined to produce a total of 153 items which took approximately 40 min to complete.

### 2.5 Ethics

Participants received an information sheet prior to giving consent and were given the opportunity to ask questions. They were informed of their withdrawal and anonymity rights. Ethical procedures aligned with British Psychological Society guidelines and the study received ethical approval from two university ethics committees in England and Spain (England Ref: FHMREC19077; Spain Ref: UV‐INV_ETICA‐1294940).

### 2.6 Procedure

Participants completed the assessments on four occasions across a 7‐month period, spanning two lockdowns. Breakdown of the date of assessments in parallel to a timeline of English and Spanish lockdowns and changing of restrictions is provided in Supplementary Material 1. Assessments were completed via telephone.

### 2.7 Statistical methods

To assess for significant change in frailty, quality of life, AFEAT, social isolation and loneliness longitudinally (Hypothesis 1) and whether any changes varied according to country (Hypothesis 2) or COVID‐19 risk level (Hypothesis 3), a series of 4 (Time) × 2 (country) × 2 (high risk, low risk) mixed‐design ANOVA were conducted. The within‐subjects factor was Time, measured at four time‐points. Age was added as a covariate for analyses. Generalised eta squared statistics (η^2^_G_) were computed as estimates of effect size. Where there were significant Time × Country, or Time × Risk interactions, post‐hoc paired‐sample *t*‐tests compared outcomes between each time point separately for the English and Spanish cohorts or separate risk groups. For all *t*‐tests, *p*‐values were adjusted using Bonferroni's correction to account for multiple comparisons.

To examine whether potential modifying factors, coping resources, physical activity, and social isolation changed over time were assessed using parametric or non‐parametric analyses as appropriate (Hypothesis 4). Then, to examine impacts of these variables on frailty, quality of life and loneliness across the time periods, a series of repeated‐measures ANCOVA were performed with Time 1 scores for total coping resources, frequency of physical activity, and social isolation as covariates as these variables may impact frailty change (Hypothesis 5).

To examine the prediction of change in frailty, quality of life, AFEAT and loneliness between strict lockdown (T1) and the period with the least restrictions (T3) on mobility and social interaction, change variables were computed between times and country, physical activity, social isolation, and coping resources entered as predictors into linear regressions (Hypothesis 5). The was to determine if coping resources, physical activity or social isolation had an impact on change in outcomes, once country and other potential confounders had been controlled for. Due to power limitations, potential confounder variables (age, education and socioeconomic status) were only included as predictors in the multiple regression models if they had significant bivariate correlations with the relevant outcome.

Sensitivity analyses were completed by imputing missing data and creating a correlation matrix between variables used in analysis and calculating root mean square error (RMSE) between the matrices. A low RMSE (~0.05) score would suggest missing data had minimal impact on analyses.

Data were analysed with SPSS Version 26 and R. Participants with missing data were excluded from analyses on a case‐wise basis.

Sample size met power requirements for analyses. Factors that may impact the effect of lockdown on frailty such as physical activity or resources used were analysed as variables of interest rather than as confounders.

## 3 RESULTS

### 3.1 Descriptive statistics

Characteristics of study participants and summary measures for frailty scores, QoL, AFEAT, loneliness and social isolation at each time point for the whole sample are presented in Table 1. At all time points, the median physical activity engagement was 30 min of exercise on 5–7 days. The average number of days between each assessment were as follows: Time 1–Time 2, Mean = 49.9, *SD* = 7.9; Time 2–Time 3, Mean = 55.1, *SD* = 7.5; Time 3–Time 4, Mean = 65.6, *SD* = 8.9.

**TABLE 1:** Descriptive statistics

Outcome	*n*	Mean	*SD*	*SE*	Interquartile range	Range
Time 1						
Frailty	70	0.280	0.11	0.01	0.13	0.083–0.532
QoL	69	35.88	6.07	0.73	8.00	22–48
AFEAT	70	33.09	5.36	0.64	6.00	19–45
Loneliness	69	9.78	2.24	0.27	3.00	4–12
Coping resources	69	4.67	1.91	0.23	3.00	0–8
Social isolation	68	42.26	14.04	1.70	11.25	16–96
Time 2						
Frailty	68	0.254	0.12	0.01	0.16	0.051–0.596
QoL	65	35.43	5.35	0.66	7.00	19–45
AFEAT	68	35.66	6.22	0.75	9.25	18–50
Loneliness	68	9.97	2.25	0.27	3.00	4–12
Coping resources	68	4.632	2.14	0.26	3.00	0–8
Social isolation	68	42.38	12.68	1.54	13.25	20–93
Time 3						
Frailty	66	0.223	0.11	0.01	0.13	0.051–0.593
QoL	64	35.61	5.21	0.65	7.00	23–46
AFEAT	66	37.09	5.99	0.74	7.75	20–49
Loneliness	66	10.17	2.12	0.26	3.00	4–12
Coping resources	63	4.81	1.58	0.20	2.00	1–8
Social isolation	66	43.64	12.28	1.51	10.75	19–89
Time 4						
Frailty	65	0.221	0.11	0.01	0.13	0.025–0.474
QoL	64	35.41	5.28	0.66	8.00	22–45
AFEAT	65	35.55	6.18	0.77	8.00	21–50
Loneliness	65	9.91	2.23	0.28	4.00	4–12
Coping resources	65	5.05	2.03	0.25	3.00	0–9
Social isolation	65	41.32	10.41	1.29	12.00	22–71

A full breakdown of individual coping resources used over time is presented in Table 2.

**TABLE 2:** Frequencies analysis showing change in use of coping resources across time points

		Time 1	Time 2	Time 3	Time 4
Family	Yes (%)	57 (81.4%)	55 (78.6%)	58 (82.9%)	57 (81.4%)
	No (%)	13 (18.6%)	13 (18.6%)	7 (10.0%)	8 (11.4%)
	Missing (%)	0	2 (2.9%)	5 (7.1%)	5 (7.1%)
Friends	Yes (%)	51 (72.9%)	51 (72.9%)	52 (74.3%)	50 (71.4%)
	No (%)	19 (27.1%)	17 (24.3%)	13 (18.6%)	15 (21.4%)
	Missing (%)	0	2 (2.9%)	5 (7.1%)	5 (7.1%)
NHS	Yes (%)	16 (22.9%)	19 (27.1%)	21 (30.0%)	22 (31.4%)
	No (%)	54 (77.1%)	49 (70.0%)	43 (61.4%)	43 (61.4%)
	Missing (%)	0	2 (2.9%)	6 (8.6%)	5 (7.1%)
Watching TV	Yes (%)	39 (55.7%)	49 (70.0%)	45 (64.3%)	54 (77.1%)
	No (%)	31 (44.3%)	19 (27.1%)	20 (28.6%)	11 (15.7%)
	Missing (%)	0	2 (2.9%)	5 (7.1%)	5 (7.1%)
Exercising	Yes (%)	42 (60.0%)	45 (64.3%)	46 (65.7%)	41 (58.6%)
	No (%)	28 (40.0%)	23 (32.9%)	19 (27.1%)	24 (34.3%)
	Missing (%)	0	2 (2.9%)	5 (7.1%)	5 (7.1%)
Community Group	Yes (%)	12 (17.1%)	16 (22.9%)	3 (4.3%)	9 (12.9%)
	No (%)	58 (82.9%)	52 (74.3%)	62 (88.6%)	56 (80.0%)
	Missing (%)	0	2 (2.9%)	5 (7.1%)	5 (7.1%)
Organisation Support	Yes (%)	23 (32.9%)	10 (14.3%)	13 (18.6%)	19 (27.1%)
	No (%)	47 (67.1%)	58 (82.9%)	52 (74.3%)	46 (65.7%)
	Missing (%)	0	2 (2.9%)	5 (7.1%)	5 (7.1%)
Food & Drink	Yes (%)	28 (40.0%)	30 (42.9%)	27 (38.6%)	35 (50.0%)
	No (%)	41 (58.6%)	38 (54.3%)	38 (54.3%)	30 (42.9%)
	Missing (%)	1 (1.4%)	2 (2.9%)	5 (7.1%)	5 (7.1%)
Humour	Yes (%)	56 (80.0%)	40 (57.1%)	44 (62.9%)	41 (58.6%)
	No (%)	14 (20.0%)	28 (40.0%)	20 (28.6%)	24 (34.3%)
	Missing (%)	0	2 (2.9%)	6 (8.6%)	5 (7.1%)

### 3.2 Sensitivity analysis

A RMSE score of 0.057 was calculated between the imputed and original data correlation matrices, indicating that the missing data had a minimal impact on output.

### 3.3 Hypotheses 1, 2 & 3: Change in outcomes over a seven month period

Results of the mixed ANOVA are presented in Table 3 (see Supplementary Material 2 for post‐hoc pairwise comparisons).

**TABLE 3:** Mixed‐design ANOVA results for frailty, QoL, AFEAT, loneliness and social isolation

Factor	*df*	*F*	*p*	Generalised η^2^
Frailty^a^				
**Country**	**1, 60**	**4.91**	**0.031**	**0.06**
**Risk**	**1, 60**	**6.47**	**0.014**	**0.07**
**Time**	**3, 180**	**9.27**	**<0.001**	**0.04**
Country * Risk	1, 60	0.19	0.665	0.00
Country * Time	**3, 180**	**2.85**	**0.039**	**0.01**
Risk * Time	3, 180	0.00	1.000	0.00
Country * Risk * Time	3, 180	0.35	0.793	0.00
QoL^b^				
Country	1, 55	1.68	0.201	0.02
Risk	1, 55	1.46	0.233	0.02
Time^c^	2.67, 146.82	0.64	0.570	0.00
Country * Risk	**1, 55**	**4.15**	**0.046**	**0.06**
Country * Time^c^	2.67, 146.82	0.46	0.691	0.00
Risk * Time^c^	2.67, 146.82	1.40	0.246	0.01
Country * Risk * Time^c^	2.67, 146.82	0.06	0.969	0.00
AFEAT^a^				
Country	1, 60	1.20	0.277	0.01
Risk	1, 60	0.00	0.995	0.00
**Time**	**3, 180**	**8.73**	**<0.001**	**0.05**
Country * Risk	1, 60	0.09	0.762	0.00
Country * Time	**3, 180**	**4.29**	**0.006**	**0.03**
Risk * Time	3, 180	0.22	0.885	0.00
Country * Risk * Time	3, 180	0.85	0.470	0.01
Loneliness^a^				
Country	1, 60	2.70	0.106	0.03
Risk	1, 60	1.39	0.244	0.02
Time	3, 180	2.65	0.050	0.01
Country * Risk	1, 60	1.46	0.231	0.02
Country * Time	3, 180	0.98	0.406	0.00
Risk * Time	3, 180	2.22	0.088	0.01
Country * Risk * Time	3, 180	2.57	0.056	0.01
Total coping resources^d^				
**Country**	**1, 57**	**55.38**	**<0.001**	**0.33**
Risk	1, 57	0.90	0.348	0.01
Time^c^	2.70, 154.12	1.65	0.186	0.01
Country * Risk	**1, 57**	**5.11**	**0.028**	**0.04**
Country * Time ^c^	**2.70, 154.12**	**3.72**	**0.016**	**0.03**
Risk * Time^c^	2.70, 154.12	1.44	0.236	0.01
Country * Risk * Time^c^	2.70, 154.12	0.42	0.717	0.00
Social isolation^e^				
Country	1, 59	2.13	0.150	0.03
Risk	1, 59	0.00	0.981	0.00
**Time**	**3, 177**	**3.81**	**0.011**	**0.02**
Country * Risk	1, 59	0.65	0.423	0.01
Country * Time	**3, 177**	**7.49**	**<0.001**	**0.03**
Risk * Time	**3, 177**	**4.55**	**0.004**	**0.02**
Country * Risk * Time	**3, 177**	**6.87**	**<0.001**	**0.03**

Note

Bold = significant at *p* < 0.05.

^a^ Six participants excluded due to missing data.

^b^ Eleven participants excluded due to missing data.

^c^ Greenhouse‐Geisser correction applied to degrees of freedom due to violation of assumption of sphericity.

^d^ Nine participants excluded due to missing data.

^e^ Seven participants excluded due to missing data.

The Spanish cohort was significantly frailer than the English cohort, and the high‐risk group were significantly frailer than the low‐risk group. A significant main effect of Time, and a significant Time x Country interaction indicated that changes in frailty over time differed between the England and Spain. Pairwise comparisons revealed that in the Spanish cohort, frailty decreased significantly from T1 to T3 (*t*(19) = −3.35, *p *< 0.05) and from T1 to T4 (*t*(19) = −4.82, *p *< 0.001). In the English cohort, frailty decreased significantly only between T1 and T3 (*t*(43) = −3.21, *p *< 0.05).

There were no significant main effects of Country or Risk for AFEAT scores. There was a significant Time effect and a Time × Country interaction. Pairwise comparisons showed that in the Spanish cohort, perceived age‐friendliness of the environment significantly increased from T1 to T2 (*t*(19) = 5.52. *p *< 0.001), T3 (*t*(19) = 6.11, *p *< 0.001), and T4 (*t*(19) = 4.53, *p *< 0.01). In the English cohort, there were significant differences between AFEAT scores at T1 compared to T3 (*t*(43) = 2.95, *p *< 0.05) and T2 compared to T3 (*t*(43) = 2.95, *p *< 0.05), again, with AFEAT increasing over time.

No significant group differences or variation over time for quality of life or loneliness were discovered.

Age did not significantly impact on change in outcomes over time.

### 3.4 Hypothesis 4: Change in potential modifying factors over time

There were no significant differences between countries or COVID‐19 risk groups for social isolation. There was a significant effect of Time and a Time × Country interaction. Pairwise comparisons showed that for the English cohort, Social Isolation score decreased significantly between T3 and T4 (*t*(42) = −2.96, *p *< 0.05), participants becoming more isolated between T3 and T4. There were no significant changes in Social Isolation over time in the Spanish cohort. There was also a significant Time × Risk interaction; however, Bonferroni corrected pairwise comparisons were all non‐significant.

The English group reported using significantly more coping resources than did the Spanish group. There was also a significant Time × Country interaction; post‐hoc comparisons revealed that the number of coping resources being used increased between T3 and T4 in the English cohort (*t*(43) = 3.35, *p *< 0.05), but there were no significant changes over time in the Spanish cohort.

There were no significant differences across time for either the Spanish or English group in exercise frequency (*χ* ^2^(3) = 5.09 and 2.29 respectively, *p* > 0.05). There was, however, a significant difference between risk groups, with the low‐risk group completing at least 30 min of exercise for more days per week than the high‐risk group (Mann Whitney *U* test).

### 3.5 Hypothesis 5: Impact of modifiers on change in frailty and well‐being over the lockdown period

The repeated‐measures ANCOVA demonstrated that the number of coping resources used did not significantly impact change in frailty (*F*(3,186) = 1.10, *p* = 0.348), loneliness (*F*(3,186) = 0.60, *p* = 0.612), AFEAT (*F*(3,186) = 1.61, *p* = 0.193), or quality of life (*F*(3,186) = 0.69, *p* = 0.545). Neither social isolation/engagement nor physical exercise significantly impacted the effect of time for loneliness, AFEAT or QoL (*p* > 0.05), and social isolation/engagement had no impact on effect of time for frailty.

Table 4 summarises the regression models predicting changes in frailty, quality of life, and loneliness between Time 1 and Time 3. Significant correlations between potential confounder variables and change scores were found between AFEAT change and age, *r* = 0.27, *p* < 0.05, and education, *r* = −0.30, *p* < 0.05. The model predicting change in AFEAT score thus included age and education as predictors. There was a trend towards a significant correlation between age and frailty change, *r* = −0.24, *p* = 0.05. No other significant correlations were observed.

**TABLE 4:** Multiple regression models predicting change in frailty, quality of life, AFEAT and loneliness and between Time 1 and Time 3

Predictor	β	*SE*	*t*	*p*
Frailty change				
Country (Spain)	−0.18	0.03	−1.22	0.226
**T1 frailty**	**−0.48**	**0.11**	**−4.16**	**<0.001**
T1 total coping resources	0.05	0.01	0.38	0.706
**T1 Physical activity**	**−0.31**	**0.01**	**−2.84**	0.**006**
**T1 social isolation**	**0.25**	**0.00**	**2.23**	0.**029**
QoL change				
Country (Spain)	−0.09	1.29	−0.61	0.541
**T1 QoL**	**−0.55**	**0.07**	**−5.09**	**<0.001**
T1 total coping resources	0.15	0.31	1.08	0.284
T1 physical activity	0.01	0.37	0.12	0.906
T1 social isolation	0.08	0.03	0.70	0.488
AFEAT change				
Age	0.08	0.16	0.65	0.516
Education	−0.06	0.74	−0.42	0.678
Country (Spain)	0.34	2.32	1.83	0.072
**T1 AFEAT**	**−0.36**	**0.13**	**−3.06**	0.**003**
T1 total coping resources	0.16	0.43	1.14	0.260
T1 physical activity	−0.01	0.54	−0.12	0.907
T1 social isolation	0.03	0.05	0.28	0.780
Loneliness Change				
Country (Spain)	0.13	0.59	0.88	0.383
**T1 Loneliness**	**−0.50**	**0.09**	**−4.35**	**<0.001**
T1 total coping resources	0.13	0.13	0.94	0.353
T1 physical activity	−0.08	0.17	−0.69	0.495
T1 social isolation	−0.02	0.02	−0.18	0.857

Note

Higher scores in the variable Social Isolation indicate lower levels of isolation. Bold = significant at *p* < 0.05.

The model predicting change in frailty was significant (*F*(5,58) = 6.66, *p* < 0.001, *R* ^2^ = 0.36, *R* ^2^_adj_ = 0.31). Frailty at Time 1 was significantly associated with the change in frailty over time. That is, those who were frailer at Time 1 experienced a greater decrease in frailty severity between Time 1 and Time 3. There was a significant association between physical activity at Time 1 and frailty change, such that those who were more physically active experienced a greater decrease in frailty. There was also a significant association between social isolation and frailty change, such that those who were less socially isolated experienced a smaller decrease in frailty over time.

The model predicting change in quality of life was also significant (*F*(5,56) = 6.05 *p* < 0.001, *R* = 0.35. *R^2^*_adj_ = 0.29). The initial level of QoL significantly predicted change, but once initial levels had been accounted for, no further variables were predictive of change.

The model predicting changes in AFEAT score was significant (*F*(7,56) = 4.03, *p* < 0.01, *R^2^* = 0.34, *R^2^*_adj_ = 0.25). AFEAT score at Time 1 significantly predicted change over time, but once initial score had been accounted for, no further variables were predictive of change.

The model predicting changes in loneliness was significant, (*F*(5,58) = 4.09, *p* < 0.01, *R* ^2^ = 0.26, *R* ^2^_adj_ = 0.20). Loneliness at Time 1 was significantly associated with the change over time, but once initial level of loneliness was accounted for, no further variables predicted change.

## 4 DISCUSSION

This study examined longitudinal changes in well‐being and COVID‐19 vulnerability using a measure of frailty, from the first pandemic related lockdown in England and Spain. The study considered whether changes differed between countries across different stages of lockdown and according to individual COVID‐19 risk based on diagnosed morbidities. We explored whether factors such as physical activity and social contact, and using coping resources mitigated adverse effects of lockdown.

Our repeated measures covered a range of lockdown severities in each country which was reflected in frailty change. Initial measures were during the most severe lockdown in each country. Spanish participants showed higher levels of frailty, perhaps related to the strictness of the Spanish lockdown, but with significant improvements with restrictions easing. Frailty severity was highest overall at the first assessment, decreasing significantly between the first and third assessments in both countries, suggesting that as restrictions eased, frailty became less severe. This trend continued in the Spanish cohort, with a significant difference between the first and fourth assessment. Conversely, average frailty increased slightly in the English cohort during the fourth assessment, and frailty was no longer significantly different compared to T1. This discrepancy suggests that restrictions being reintroduced prior to the fourth assessment were having differing effects: in England, regional tier‐based restrictions were introduced. North West England had stronger restrictions than other regions, which may explain the reversal in the reduction of frailty severity, underlining the impact of lockdown vulnerability. The tendency for frailty to be more severe when lockdown restrictions are at their highest is a notable finding, given that frailty is a significant risk factor for severe disease outcomes and mortality as a result of COVID‐19 (Hewitt et al., 2020; Ma et al., 2020; Petermann‐Rocha et al., 2020). Frailty also impacts on vulnerability to non‐COVID‐19 related adverse outcomes (Morley, 2013), which in a time of depleted health resources may have serious outcomes for individuals. It should be noted, that as the present study did not include a measure of frailty before the pandemic started, we cannot conclude that lockdown directly caused an increase in frailty, but does suggest that while lockdowns reduce the risk of contracting COVID‐19, they increase vulnerability to adverse outcomes.

Perceptions of environmental age‐friendliness (AFEAT) also varied over time and between countries. In the Spanish cohort, AFEAT increased earlier, at the second assessment. This reflects earlier changes in lockdown restrictions in Spain. In England, participants perceived their environment to be more age‐friendly at the time of the third assessment, when lockdown restrictions were at their lowest. However, by the time of the fourth assessment, restrictions had been reintroduced in England, and the difference from Time 1 was no longer significant. The importance of the environment would be expected to vary with lockdowns as the measure includes items relating to accessibility of services and mobility within the community, and items related to community outreach to include people at risk of social isolation, or feeling a valuable part of one's local community. Previous work has shown that this measure acts as an important moderator of the relationship between frailty and need for formal care (Garner & Holland, 2019), and the variation with lockdown underlines areas for intervention in future lockdowns. This is demonstrated in the effects of lockdown variation on the measure of social isolation used, with a reduction in this measure (indicating more isolation) for the participants in England as a second wave of lockdowns, particularly in areas of the North West of England, began to have effect at Time 4.

There was, however, little evidence that loneliness changed significantly over time. Previous research from both the UK and Spain has shown that younger adults may be more at risk of loneliness during lockdown (Bu et al., 2020; Losada‐Baltar et al., 2021), possibly because younger people normally lead more active social lifestyles outside the home and would be at work or places of education and therefore experienced more social disruption due to lockdown measures. The findings reported here support this; social isolation levels among older adults only changed at Time 4, and there was no change in feelings of loneliness. Our parallel qualitative work in the England with some of the same participants (Varey et al., in prep) has shown that families and friends made a significant effort to stay in contact during the first lockdown and mood was generally resilient and positive, but by the time of the increasing lockdown restrictions at Time 4, this positivity was giving way to anxiety about how long such isolation would continue for.

To the best of our knowledge, no studies have examined longitudinal changes to the quality of life during the COVID‐19 pandemic, although one study did report a significant decrease compared to pre‐pandemic levels in a sample of older adults (Rantanen et al., 2021) and another study demonstrated negative impact of lockdown longitudinally on health and social well‐being of clinically vulnerable older adults (Di Gessa & Price, 2021). While we cannot rule out the possibility that quality of life in our sample may have been higher before the pandemic, there were no significant changes to the quality of life over time. That is, in both England and Spain, our older adult sample's quality of life was notably stable throughout periods of lockdown.

Another notable finding is the lack of significant differences between those who were and were not considered high risk. While frailty was significantly more severe in the high‐risk group as one may expect, there were no differences in quality of life, AFEAT, social isolation or loneliness. One might have expected those who were at greater risk from COVID‐19 to experience negative effects of lockdown more acutely, but this is not apparent. This contrasts with the findings of Di Gessa and Price (2021) indicated above, although they only compared clinically vulnerable with non‐clinically vulnerable older adults (controlling for pre‐pandemic levels) on assessments that took place during June/July 2020 (using data from the English Longitudinal Study of Ageing, ELSA). Our parallel qualitative work suggests an explanation for our findings (Varey et al., in prep)–that those living with significant chronic diseases were already living somewhat restricted lives and so lockdown made less difference to them than to their more active healthy counterparts. It is possible that older adults may have behaved similarly regardless of their risk‐level, but more research regarding the physical and psychological effects among those shielding is needed to explore this possibility further.

The majority of the sample reported using social support as a coping resource throughout the study, which may explain the lack of significant improvement in feelings of loneliness and social isolation once lockdown restrictions had eased. Overall, the English group reported using more coping resources than the Spanish group. This may suggest that such coping resources were less available to the Spanish group during lockdown periods. Further studies should explore whether the Spanish older adults had poorer access to or less knowledge of alternative coping resources or new technologies compared to the English population.

Our analysis of the hypothesised contributory covariates showed that social engagement/isolation, physical activity or number of coping resources used had no impact on changes in quality of life, environmental perceptions and loneliness. However, in relation to changes in frailty, regression analyses showed that physical activity and social engagement/isolation at T1 had significant impacts on change in frailty once contributions of country and initial frailty levels had been accounted for. Those who were more physically active at the time of the first assessment showed greater decreases in frailty severity between the first and third assessments, in line with evidence that high levels of physical activity are linked to reduced risk of frailty (Oliveira et al., 2020). This is important and supports the hypothesis that continued physical activity during lockdown could modify changes in frailty and related vulnerability to severe COVID‐19 reactions. These findings highlight the importance of maintaining sufficient exercise levels during lockdown and limiting exercise to reduce the spread of COVID‐19 will negatively impact on older adults.

### 4.1 Limitations

The differences between T1 and T3 in outcomes indicated that lockdown affected health and well‐being. However, as we have no ‘pre‐COVID’ assessment, we cannot confirm the full extent of the effects of lockdown, which may be more severe than analyses suggest.

The sample size, while considered sufficient through power analysis, remains relatively small and therefore generalisability may be limited.

## 5 CONCLUSIONS

The study examined changes to health and well‐being in England and Spain during the COVID‐19 pandemic. Results showed similar trends in both countries; as lockdown restrictions were eased, frailty severity decreased, and older adults viewed their environment as being more age‐friendly, particularly in Spain where initial lockdown was more severe; differences between the countries seemed to be related to different lockdowns. Changes in perceived environmental age‐friendliness is important as it moderates the impact of frailty on social care requirements and quality of life (Garner & Holland, 2019). Social isolation remained largely unchanged except for when English restrictions were re‐introduced. There were no significant changes in loneliness or quality of life. Being frailer, more physically active and more socially isolated initially was associated with greater decreases in frailty as lockdowns eased. Addressing physical activity and social isolation should be carefully promoted to mitigate impact on frailty of future lockdowns.

## CONFLICT OF INTEREST

None.

## AUTHOR CONTRIBUTIONS

C. A. Holland and S. Varey developed the research concept. C. A. Holland, S. Varey, and I. W. Garner designed the study. I. W. Garner, S. Varey, E. Navarro‐Pardo and C. Marr collected the data. I. W. Garner and C. Marr analysed the data. I. W. Garner, C. A. Holland and C. Marr wrote the manuscript. All authors reviewed and approved the final manuscript for submission.

## Supporting information

Supplementary Material

Supplementary Material

## Data Availability

Data will not be made publicly available yet as analyses are ongoing with the aim of additional publications.

## ENDNOTES

^1^
Analyses on frailty exclude physical activity as a covariate because physical activity is part of the frailty assessment.

^2^
‘Shielding’ is an instruction to avoid all physical contact with others.

## References

[hsc13735-bib-0001] Apóstolo, J. , Cooke, R. , Bobrowicz‐Campos, E. , Santana, S. , Marcucci, M. , Cano, A. , Vollenbroek‐Hutten, M. , Germini, F. , D’Avanzo, B. , Gwyther, H. , & Holland, C. (2018). Effectiveness of interventions to prevent pre‐frailty and frailty progression in older adults: A systematic review. JBI Database of Systematic Reviews and Implementation Reports, 16(1), 140–232. 10.11124/JBISRIR-2017-003382 29324562 PMC5771690

[hsc13735-bib-0002] Atkins, J. L. , Masoli, J. A. H. , Delgado, J. , Pilling, L. C. , Kuo, C.‐L. , Kuchel, G. A. , & Melzer, D. (2020). Preexisting comorbidities predicting COVID‐19 and mortality in the UK Biobank Community Cohort. The Journals of Gerontology: Series A, 75(11), 2224–2230. 10.1093/gerona/glaa183 PMC7454409 32687551

[hsc13735-bib-0003] Bell, C. , Fausset, C. , Farmer, S. , Nguyen, J. , Harley, L. , & Fain, W. B. (2013, May). Examining social media use among older adults. In Proceedings of the 24th ACM conference on hypertext and social media (pp. 158–163). 10.1145/2481492.2481509

[hsc13735-bib-0004] Bu, F. , Steptoe, A. , & Fancourt, D. (2020). Loneliness during a strict lockdown: Trajectories and predictors during the COVID‐19 pandemic in 38,217 United Kingdom adults. Social Science & Medicine, 265, 113521. 10.1016/j.socscimed.2020.113521 PMC7768183 33257177

[hsc13735-bib-0006] Carriedo, A. , Cecchini, J. A. , Fernandez‐Rio, J. , & Mendez‐Gimenez, A. (2020). COVID‐19, psychological well‐being and physical activity levels in older adults during the nationwide lockdown in Spain. The American Journal of Geriatric Psychiatry, 28(11), 1146–1155. 10.1016/j.jagp.2020.08.007 32919872 PMC7443087

[hsc13735-bib-0009] Clegg, A. , Young, J. , Iliffe, S. , Rikkert, M. O. , & Rockwood, K. (2013). Frailty in elderly people. The Lancet, 381(9868), 752–762. 10.1016/S0140-6736(12)62167-9 PMC4098658 23395245

[hsc13735-bib-0010] Di Gessa, G. , & Price, D. (2021). Changes in health and social well‐being in the COVID‐19 clinically vulnerable older English population during the pandemic. Journal of Epidemiology Community Health, 75(11), 1070–1077. 10.1136/jech-2021-216405 33947747 PMC8103553

[hsc13735-bib-0050] Fang, Y. , Chau, A. K. , Wong, A. , Fung, H. H. , & Woo, J. (2018). Information and communicative technology use enhances psychological well‐being of older adults: the roles of age, social connectedness, and frailty status. Aging & Mental Health, 22(11), 1516–1524. 28777010 10.1080/13607863.2017.1358354

[hsc13735-bib-0011] Gale, C. R. , Westbury, L. , & Cooper, C. (2018). Social isolation and loneliness as risk factors for the progression of frailty: The English Longitudinal Study of Ageing. Age and Ageing, 47(3), 392–397. 10.1093/ageing/afx188 29309502 PMC5920346

[hsc13735-bib-0012] García‐Fernández, L. , Romero‐Ferreiro, V. , López‐Roldán, P. D. , Padilla, S. , & Rodriguez‐Jimenez, R. (2020). Mental health in elderly Spanish people in times of Covid‐19 outbreak. The American Journal of Geriatric Psychiatry, 28(10), 1040–1045. 10.1016/j.jagp.2020.06.027 32718855 PMC7340042

[hsc13735-bib-0013] Garner, I. W. , Burgess, A. P. , & Holland, C. A. (2020). Developing and validating the Community‐Oriented Frailty Index (COM‐FI). Archives of Gerontology and Geriatrics, 91, 10.1016/j.archger.2020.104232. 104232 32827944

[hsc13735-bib-0014] Garner, I. W. , & Holland, C. A. (2019). Age‐friendliness of living environments from the older person’s viewpoint: Development of the Age‐Friendly Environment Assessment Tool. Age and Ageing, 49(2), 193–198. 10.1093/ageing/afz146 31790132

[hsc13735-bib-0015] Gobbens, R. J. , van Assen, M. A. , Luijkx, K. G. , & Schols, J. M. (2012). The predictive validity of the Tilburg Frailty Indicator: Disability, health care utilization, and quality of life in a population at risk. The Gerontologist, 52(5), 619–631. 10.1093/geront/gnr135 22217462

[hsc13735-bib-0016] Gombault, V. (2013). The internet more and more popular, the internet user more and more mobile. Insee Premiere, 1452, 1–4. http://www.epsilon.insee.fr/jspui/bitstream/1/17875/1/ip1452.pdf

[hsc13735-bib-0017] González‐Sanguino, C. , Ausín, B. , Castellanos, M. A. , Saiz, J. , López‐Gómez, A. , Ugidos, C. , & Muñoz, M. (2020). Mental health consequences during the initial stage of the 2020 Coronavirus pandemic (COVID‐19) in Spain. Brain, Behavior, and Immunity, 87, 172–176. 10.1016/j.bbi.2020.05.040 32405150 PMC7219372

[hsc13735-bib-0019] Hewitt, J. , Carter, B. , Vilches‐Moraga, A. , Quinn, T. J. , Braude, P. , Verduri, A. , Pearce, L. , Stechman, M. , Short, R. , Price, A. , Collins, J. T. , Bruce, E. , Einarsson, A. , Rickard, F. , Mitchell, E. , Holloway, M. , Hesford, J. , Barlow‐Pay, F. , Clini, E. , … Guaraldi, G. (2020). The effect of frailty on survival in patients with COVID‐19 (COPE): A multicentre, European, observational cohort study. The Lancet Public Health, 5(8), e444–e451. 10.1016/S2468-2667(20)30146-8 32619408 PMC7326416

[hsc13735-bib-0021] Levin, A. T. , Hanage, W. P. , Owusu‐Boaitey, N. , Cochran, K. B. , Walsh, S. P. , & Meyerowitz‐Katz, G. (2020). Assessing the age specificity of infection fatality rates for COVID‐19: Systematic review, meta‐analysis, and public policy implications. European Journal of Epidemiology, 35(12), 1123–1138. 10.1007/s10654-020-00698-1 33289900 PMC7721859

[hsc13735-bib-0023] Livingston, G. , Huntley, J. , Sommerlad, A. , Ames, D. , Ballard, C. , Banerjee, S. , Brayne, C. , Burns, A. , Cohen‐Mansfield, J. , Cooper, C. , Costafreda, S. G. , Dias, A. , Fox, N. , Gitlin, L. N. , Howard, R. , Kales, H. C. , Kivimäki, M. , Larson, E. B. , Ogunniyi, A. , … Mukadam, N. (2020). Dementia prevention, intervention, and care: 2020 report of the Lancet Commission. The Lancet, 396(10248), 413–446. 10.1016/S0140-6736(20)30367-6 PMC7392084 32738937

[hsc13735-bib-0025] Losada‐Baltar, A. , Jiménez‐Gonzalo, L. , Gallego‐Alberto, L. , Pedroso‐Chaparro, M. D. S. , Fernandes‐Pires, J. , & Márquez‐González, M. (2021). “We are staying at home”. Association of self‐perceptions of aging, personal and family resources, and loneliness with psychological distress during the lock‐down period of COVID‐19. The Journals of Gerontology: Series B, 76(2), e10–e16. 10.1093/geronb/gbaa048 PMC7184373 32282920

[hsc13735-bib-0026] Ma, Y. , Hou, L. , Yang, X. , Huang, Z. , Yang, X. , Zhao, N. A. , He, M. , Shi, Y. , Kang, Y. , Yue, J. , & Wu, C. (2020). The association between frailty and severe disease among COVID‐19 patients aged over 60 years in China: A prospective cohort study. BMC Medicine, 18(1), 274. 10.1186/s12916-020-01761-0 32892742 PMC7474968

[hsc13735-bib-0028] Morley, J. E. (2013). Frailty: A time for action. European Geriatric Medicine, 4(4), 215–216. 10.1016/j.eurger.2013.08.006

[hsc13735-bib-0030] Netten, A. , Burge, P. , Malley, J. , Potoglou, D. , Towers, A. M. , Brazier, J. , Flynn, T. , Forder, J. , & Wall, B. (2012). Outcomes of social care for adults: Developing a preference‐weighted measure. In T. Walley , M. Ashton‐Key , A. Clarke , T. Marshall , J. Powell , R. Riemsma , K. Stein , & P. Davidson (Eds.), NIHR Health Technology Assessment Programme: Executive Summaries (pp. 1–166). NIHR Journals Library. 10.3310/hta16160 22459668

[hsc13735-bib-0031] NHS . (2020). Who is at high risk from coronavirus (clinically extremely vulnerable). https://www.nhs.uk/conditions/coronavirus‐covid‐19/people‐at‐higher‐risk/who‐is‐at‐high‐risk‐from‐coronavirus‐clinically‐extremely‐vulnerable/

[hsc13735-bib-0033] Oliveira, J. S. , Pinheiro, M. B. , Fairhall, N. , Walsh, S. , Franks, T. C. , Kwok, W. , Bauman, A. , & Sherrington, C. (2020). Evidence on physical activity and the prevention of frailty and sarcopenia among older people: A systematic review to inform the World Health Organization Physical Activity Guidelines. Journal of Physical Activity and Health, 17(12), 1247–1258. 10.1123/jpah.2020-0323 32781432

[hsc13735-bib-0034] Onder, G. , Rezza, G. , & Brusaferro, S. (2020). Case‐fatality rate and characteristics of patients dying in relation to COVID‐19 in Italy. Journal of the American Medical Association, 323(18), 1775–1776. 10.1001/jama.2020.4683 32203977

[hsc13735-bib-0035] Parrado‐Gonzalez, A. , & León‐Jariego, J. (2020). COVID‐19: Factors associated with emotional distress and psychological morbidity in Spanish population. Revista Española de Salud Pública, 94, e202006058. 32507849 PMC11583131

[hsc13735-bib-0036] Petermann‐Rocha, F. , Hanlon, P. , Gray, S. R. , Welsh, P. , Gill, J. M. R. , Foster, H. , Katikireddi, S. V. , Lyall, D. , Mackay, D. F. , O’Donnell, C. A. , Sattar, N. , Nicholl, B. I. , Pell, J. P. , Jani, B. D. , Ho, F. K. , Mair, F. S. , & Celis‐Morales, C. (2020). Comparison of two different frailty measurements and risk of hospitalisation or death from COVID‐19: Findings from UK Biobank. BMC Medicine, 18(1), 355. 10.1186/s12916-020-01822-4 33167965 PMC7652674

[hsc13735-bib-0037] Picaza‐Gorrochategi, M. , Eiguren Munitis, A. , Dosil Santamaria, M. , & Ozamiz Etxebarria, N. (2020). Stress, anxiety, and depression in people aged over 60 in the COVID‐19 outbreak in a sample collected in Northern Spain. The American Journal of Geriatric Psychiatry, 28(9), 993–998. 10.1016/j.jagp.2020.05.022 32576424 PMC7261426

[hsc13735-bib-0038] Pierce, M. , Hope, H. , Ford, T. , Hatch, S. , Hotopf, M. , John, A. , Kontopantelis, E. , Webb, R. , Wessely, S. , McManus, S. , & Abel, K. M. (2020). Mental health before and during the COVID‐19 pandemic: A longitudinal probability sample survey of the UK population. Lancet Psychiatry, 7(10), 883–892. 10.1016/S2215-0366(20)30308-4 32707037 PMC7373389

[hsc13735-bib-0039] Planchuelo‐Gómez, A. , Odriozola‐González, P. , Irurtia, M. J. , & de Luis‐García, R. (2020). Longitudinal evaluation of the psychological impact of the COVID‐19 crisis in Spain. Journal of Affective Disorders, 277, 842–849. 10.1016/j.jad.2020.09.018 33065825 PMC7476580

[hsc13735-bib-0040] Rantanen, T. , Eronen, J. , Kauppinen, M. , Kokko, K. , Sanaslahti, S. , Kajan, N. , & Portegijs, E. (2021). Life‐space mobility and active aging as factors underlying quality of life among older people before and during COVID‐19 lockdown in Finland—A longitudinal study. The Journals of Gerontology: Series A, 76(3), e60–e67. 10.1093/gerona/glaa274 PMC7665359 33125043

[hsc13735-bib-0041] Rodríguez‐Gonzalez, R. , Facal, D. , Martinez‐Santos, A.‐E. , & Gandoy‐Crego, M. (2020). Psychological, social and health‐related challenges in Spanish older adults during the lockdown of the COVID‐19 first wave. Frontiers in Psychiatry, 11, 588949. 10.3389/fpsyt.2020.588949 PMC7744409 33343421

[hsc13735-bib-0042] Rossi, R. , Jannini, T. B. , Socci, V. , Pacitti, F. , & Di Lorenzo, G. (2021). Stressful life events and resilience during the COVID‐19 lockdown measures in Italy: Association with mental health outcomes and age. Frontiers in Psychiatry, 12, 635832. 10.3389/fpsyt.2021.635832 PMC7982412 33762980

[hsc13735-bib-0044] Russell, D. , Peplau, L. A. , & Cutrona, C. E. (1980). The revised UCLA Loneliness Scale: Concurrent and discriminant validity evidence. Journal of Personality and Social Psychology, 39(3), 472–480. 10.1037//0022-3514.39.3.472 7431205

[hsc13735-bib-0045] The National Statistics Socio‐Economic Classification. (2005). https://www.ons.gov.uk/methodology/classificationsandstandards/otherclassifications/thenationalstatisticssocioeconomicclassificationnssecrebasedonsoc2010

[hsc13735-bib-0046] von Elm, E. , Altman, D. G. , Egger, M. , Pocock, S. J. , Gøtzsche, P. C. , Vandenbroucke, J. P. , & STROBE Initiative . (2007). The Strengthening the Reporting of Observational Studies in Epidemiology (STROBE) statement: Guidelines for reporting observational studies. PLoS Medicine, 4(10), e296. 10.1371/journal.pmed.0040296 17941714 PMC2020495

[hsc13735-bib-0048] Wiggins, R. , Netuveli, G. , Hyde, M. , Higgs, P. , & Blane, D. (2008). The evaluation of a self‐enumerated scale of quality of life (CASP 19) in the context of research on ageing: A combination of explanatory and confirmatory approaches. Social Indicators Research, 89, 61–77. 10.1007/s11136-010-9835-x

[hsc13735-bib-0049] Williamson, E. J. , Walker, A. J. , Bhaskaran, K. , Bacon, S. , Bates, C. , Morton, C. E. , Curtis, H. J. , Mehrkar, A. , Evans, D. , Inglesby, P. , Cockburn, J. , McDonald, H. I. , MacKenna, B. , Tomlinson, L. , Douglas, I. J. , Rentsch, C. T. , Mathur, R. , Wong, A. Y. S. , Grieve, R. , … Goldacre, B. (2020). Factors associated with COVID‐19‐related death using openSAFELY. Nature, 584, 430–436. 10.1038/s41586-020-2521-4 32640463 PMC7611074

