# Supplementary figures and images for: An observational cohort study of longitudinal impacts on frailty and well‐being of COVID‐19 lockdowns in older adults in England and Spain
Source: Health Soc Care Community. 2022 Jan 28;30(5):e2905–16. doi: 10.1111/hsc.13735 (PMC9545919; doi:10.1111/hsc.13735)

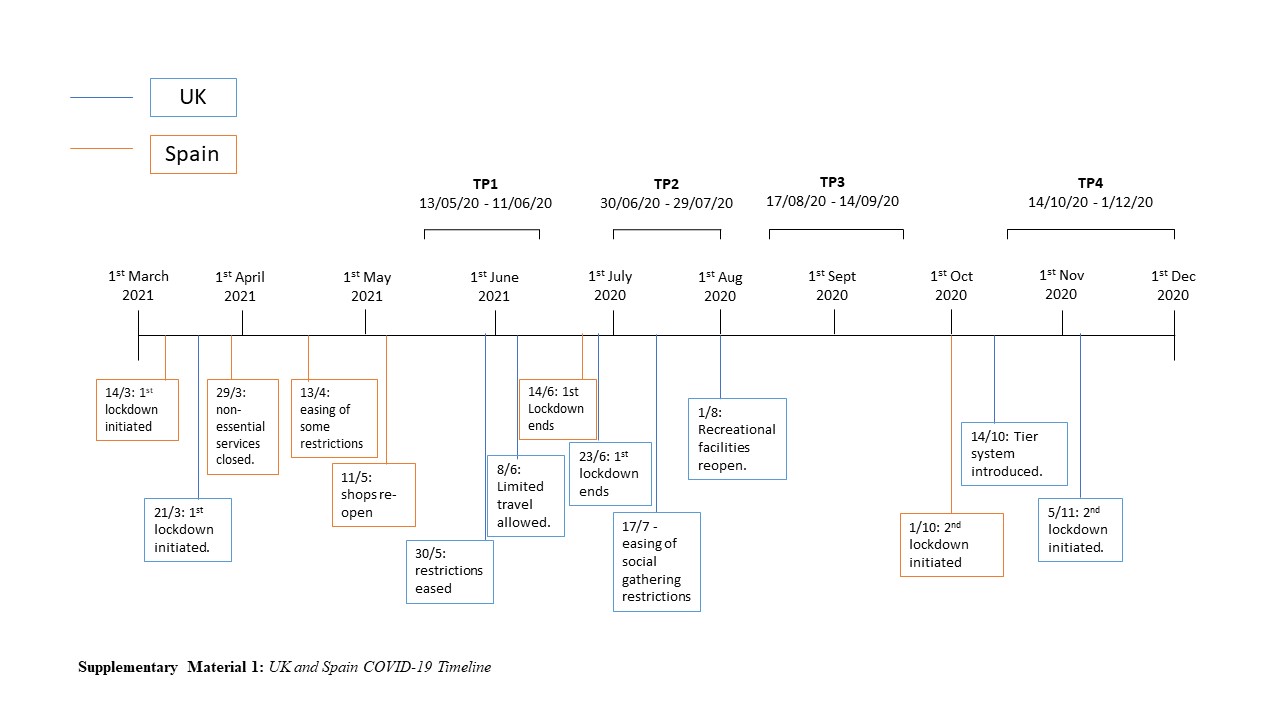

Supplement: Supplementary file 1 — Supplementary Material [file HSC-30-e2905-s002.jpg]
