# Supplementary material for: An observational cohort study of longitudinal impacts on frailty and well‐being of COVID‐19 lockdowns in older adults in England and Spain
Source: Health Soc Care Community. 2022 Jan 28;30(5):e2905–16. doi: 10.1111/hsc.13735 (PMC9545919; doi:10.1111/hsc.13735)
Supplement: Supplementary file 2 — Supplementary Material [file HSC-30-e2905-s001.docx]

**Supplementary Material 2**

**Table S1:** *Means and Standard Deviations of Study Variables in the English and Spanish Cohorts*

| Outcome | England | | |  | Spain | | |
| --- | --- | --- | --- | --- | --- | --- | --- |
|  | n | Mean | Std. Dev. |  | n | Mean | Std. Dev. |
| Time 1 |  |  |  |  |  |  |  |
| Frailty | 50 | 0.259 | 0.10 |  | 20 | 0.334 | 0.11 |
| QoL | 49 | 35.86 | 6.03 |  | 20 | 35.95 | 6.34 |
| AFEAT | 50 | 33.82 | 5.52 |  | 20 | 31.25 | 4.56 |
| Loneliness | 49 | 9.47 | 2.42 |  | 20 | 10.55 | 1.54 |
| Social Isolation | 48 | 39.25 | 9.34 |  | 20 | 49.50 | 20.02 |
| Coping Resources | 49 | 5.37 | 1.60 |  | 20 | 2.95 | 1.50 |
| Time 2 |  |  |  |  |  |  |  |
| Frailty | 48 | 0.242 | 0.11 |  | 20 | 0.282 | 0.12 |
| QoL | 45 | 35.42 | 5.11 |  | 20 | 35.45 | 6.01 |
| AFEAT | 48 | 34.06 | 5.69 |  | 20 | 39.50 | 5.85 |
| Loneliness | 48 | 9.54 | 2.43 |  | 20 | 11.00 | 1.30 |
| Social Isolation | 48 | 39.67 | 9.32 |  | 20 | 48.90 | 16.99 |
| Coping Resources | 48 | 5.56 | 1.50 |  | 20 | 2.40 | 1.76 |
| Time 3 |  |  |  |  |  |  |  |
| Frailty | 46 | 0.216 | 0.11 |  | 20 | 0.238 | 0.11 |
| QoL | 44 | 36.02 | 5.21 |  | 20 | 34.70 | 5.24 |
| AFEAT | 46 | 36.33 | 6.13 |  | 20 | 38.85 | 5.38 |
| Loneliness | 46 | 9.87 | 2.23 |  | 20 | 10.85 | 1.73 |
| Social Isolation | 46 | 42.50 | 11.95 |  | 20 | 46.25 | 12.93 |
| Coping Resources | 46 | 5.24 | 1.42 |  | 17 | 3.65 | 1.46 |
| Time 4 |  |  |  |  |  |  |  |
| Frailty | 45 | 0.218 | 0.11 |  | 20 | 0.230 | 0.10 |
| QoL | 45 | 35.44 | 5.17 |  | 19 | 35.32 | 5.69 |
| AFEAT | 45 | 34.73 | 6.15 |  | 20 | 37.40 | 5.98 |
| Loneliness | 45 | 9.42 | 2.40 |  | 20 | 11.00 | 1.30 |
| Social Isolation | 45 | 39.38 | 9.22 |  | 20 | 45.70 | 11.81 |
| Coping Resources | 45 | 5.89 | 1.56 |  | 20 | 3.15 | 1.69 |

**Table S2:** *Means and Standard Deviations of Study Variables in those at High Risk and Low Risk from COVID-19*

| Outcome | Low Risk | | |  | High Risk | | |
| --- | --- | --- | --- | --- | --- | --- | --- |
|  | n | Mean | Std. Dev. |  | n | Mean | Std. Dev. |
| Time 1 |  |  |  |  |  |  |  |
| Frailty | 46 | 0.261 | 0.10 |  | 24 | 0.318 | 0.11 |
| QoL | 45 | 36.42 | 6.52 |  | 24 | 34.88 | 5.11 |
| AFEAT | 46 | 33.09 | 5.86 |  | 24 | 33.08 | 4.37 |
| Loneliness | 46 | 9.57 | 2.39 |  | 23 | 10.22 | 1.88 |
| Social Isolation | 45 | 42.42 | 13.82 |  | 23 | 41.96 | 14.76 |
| Coping Sources | 46 | 4.52 | 1.95 |  | 23 | 4.96 | 1.85 |
| Time 2 |  |  |  |  |  |  |  |
| Frailty | 45 | 0.233 | 0.11 |  | 23 | 0.296 | 0.13 |
| QoL | 42 | 35.00 | 5.42 |  | 23 | 36.22 | 5.26 |
| AFEAT | 45 | 36.27 | 6.41 |  | 23 | 34.48 | 5.78 |
| Loneliness | 45 | 9.84 | 2.48 |  | 23 | 10.22 | 1.76 |
| Social Isolation | 45 | 42.98 | 13.80 |  | 23 | 41.22 | 10.31 |
| Coping Sources | 45 | 4.31 | 2.15 |  | 23 | 5.26 | 2.00 |
| Time 3 |  |  |  |  |  |  |  |
| Frailty | 46 | 0.199 | 0.10 |  | 20 | 0.279 | 0.13 |
| QoL | 45 | 35.71 | 5.46 |  | 19 | 35.37 | 4.72 |
| AFEAT | 46 | 37.41 | 6.22 |  | 20 | 36.35 | 5.48 |
| Loneliness | 46 | 9.87 | 2.32 |  | 20 | 10.85 | 1.42 |
| Social Isolation | 46 | 44.39 | 13.41 |  | 20 | 41.90 | 9.23 |
| Coping Sources | 44 | 4.55 | 1.56 |  | 19 | 5.42 | 1.50 |
| Time 4 |  |  |  |  |  |  |  |
| Frailty | 46 | 0.201 | 0.09 |  | 19 | 0.272 | 0.13 |
| QoL | 45 | 35.53 | 5.21 |  | 19 | 35.11 | 5.60 |
| AFEAT | 46 | 35.59 | 6.99 |  | 19 | 35.47 | 3.67 |
| Loneliness | 46 | 9.61 | 2.39 |  | 19 | 10.63 | 1.64 |
| Social Isolation | 46 | 41.17 | 11.37 |  | 19 | 41.68 | 7.88 |
| Coping Sources | 46 | 4.91 | 2.10 |  | 19 | 5.37 | 1.89 |

**Table S3:** *Pairwise Comparisons of Frailty Scores Over Time for the English and Spanish Cohorts*

| Comparison | England (N = 44) | |  | Spain (N = 20) | |
| --- | --- | --- | --- | --- | --- |
|  | Mean Difference | t |  | Mean Difference | t |
| Time 2 – Time 1 | -0.020 | -1.87 |  | -0.052 | -2.22 |
| Time 3 – Time 1 | -0.039 | -3.21* |  | -0.096 | -3.35* |
| Time 4 – Time 1 | -0.031 | -2.62 |  | -0.104 | -4.82*** |
| Time 3 – Time 2 | -0.019 | -1.58 |  | -0.044 | -1.87 |
| Time 4 – Time 2 | -0.012 | -0.96 |  | -0.052 | -2.66 |
| Time 4 – Time 3 | 0.008 | 0.57 |  | -0.008 | -0.53 |

*Note*. All *p* values are Bonferroni corrected for six comparisons.

**p* < .05. ***p* < .01. ****p* < .001

**Table S4**

*Pairwise Comparisons of AFEAT Scores Over Time for the English and Spanish Cohorts*

| Comparison | England (N = 44) | |  | Spain (N = 20) | |
| --- | --- | --- | --- | --- | --- |
|  | Mean Difference | t |  | Mean Difference | t |
| Time 2 – Time 1 | 0.36 | 0.53 |  | 8.25 | 5.52*** |
| Time 3 – Time 1 | 2.39 | 2.95* |  | 7.60 | 6.11*** |
| Time 4 – Time 1 | 0.64 | 0.78 |  | 6.15 | 4.53** |
| Time 3 – Time 2 | 2.02 | 2.95* |  | -0.65 | -0.39 |
| Time 4 – Time 2 | 0.27 | 0.45 |  | -2.10 | -0.92 |
| Time 4 – Time 3 | -1.75 | -2.60 |  | -1.45 | -1.16 |

*Note*. All *p* values are Bonferroni corrected for six comparisons.

**p* < .05. ***p* < .01. ****p* < .001

**Table S5:** *Pairwise Comparisons of Social Isolation Scores Over Time for the English and Spanish Cohorts*

| Comparison | England (N = 43) | |  | Spain (N = 20) | |
| --- | --- | --- | --- | --- | --- |
|  | Mean Difference | t |  | Mean Difference | t |
| Time 2 – Time 1 | 1.05 | 0.94 |  | -0.60 | -0.15 |
| Time 3 – Time 1 | 3.65 | 2.43 |  | -3.25 | -1.05 |
| Time 4 – Time 1 | -0.26 | -0.23 |  | -3.80 | -1.18 |
| Time 3 – Time 2 | 2.60 | 1.79 |  | -2.65 | -1.06 |
| Time 4 – Time 2 | -1.30 | -1.20 |  | -3.20 | -1.23 |
| Time 4 – Time 3 | -3.91 | -2.96* |  | -0.55 | -0.42 |

*Note*. All *p* values are Bonferroni corrected for six comparisons.

**p* < .05. ***p* < .01. ****p* < .001

**Table S6:** *Pairwise Comparisons of Social Isolation Scores Over Time for the Low Risk and High Risk Groups*

| Comparison | Low Risk (N = 44) | |  | High Risk (N = 19) | |
| --- | --- | --- | --- | --- | --- |
|  | Mean Difference | t |  | Mean Difference | t |
| Time 2 – Time 1 | 0.70 | 0.47 |  | 0.11 | 0.03 |
| Time 3 – Time 1 | 2.27 | 1.44 |  | -0.42 | -0.13 |
| Time 4 – Time 1 | -1.18 | -0.87 |  | -1.84 | -0.64 |
| Time 3 – Time 2 | 1.57 | 0.94 |  | -0.53 | -0.28 |
| Time 4 – Time 2 | -1.89 | -1.46 |  | -1.95 | -0.91 |
| Time 4 – Time 3 | -3.45 | -2.73 |  | -1.42 | -0.89 |

Note. All *p* values are Bonferroni corrected for six comparisons.

**p* < .05. ***p* < .01. ****p* < .001

**Table S7:** *Pairwise Comparisons of Total Coping Resources Used Over Time for the English and Spanish Cohorts*

| Comparison | Low Risk (N = 44) | |  | High Risk (N = 19) | |
| --- | --- | --- | --- | --- | --- |
|  | Mean Difference | t |  | Mean Difference | t |
| Time 2 – Time 1 | 0.11 | 0.45 |  | -0.59 | -0.98 |
| Time 3 – Time 1 | -0.23 | -0.82 |  | 0.76 | 1.92 |
| Time 4 – Time 1 | 0.41 | 1.59 |  | 0.24 | 0.46 |
| Time 3 – Time 2 | -0.34 | -1.68 |  | 1.35 | 2.41 |
| Time 4 – Time 2 | 0.30 | 1.32 |  | 0.82 | 1.33 |
| Time 4 – Time 3 | 0.64 | 3.35* |  | -0.53 | -1.34 |

Note. All *p* values are Bonferroni corrected for six comparisons.

**p* < .05. ***p* < .01. ****p* < .001
